# Different suture anchor fixation techniques affect contact properties in humeral greater tuberosity fracture: a biomechanical study

**DOI:** 10.1186/s12891-019-2412-8

**Published:** 2019-01-17

**Authors:** Cheng-Li Lin, Ming-Long Yeh, Fong-Chin Su, Yu-Chih Wang, Chen-Hao Chiang, Chih-Kai Hong, Wei-Ren Su

**Affiliations:** 1 Department of Orthopaedic Surgery, National Cheng Kung University Hospital, College of Medicine, National Cheng Kung University, No.138, Sheng-Li Road, 70428, Tainan City, Taiwan; 2 Department of Biomedical Engineering, National Cheng Kung University, Tainan, Taiwan; 3 Medical Device R & D Core Laboratory, National Cheng Kung University Hospital, Tainan, Taiwan; 4 Medical Device Innovation Center, National Cheng Kung University, Tainan, Taiwan; 5 Department of Orthopaedics, Chiayi Christian Hospital, Chiayi, Taiwan

**Keywords:** Humeral greater tuberosity fracture, Suture anchor, Double-row, Contact pressure, Contact area, Abduction

## Abstract

**Background:**

Suture anchor-based fixations of humeral greater tuberosity (GT) fractures have yielded good outcomes in both clinical and biomechanical studies. Be that as it may, the interface contact properties of these fixations have yet to be elaborated. In response, the contact characteristics of two double-row suture anchor fixations for the management of GT fracture were compared.

**Methods:**

Two suture anchor-based fixation techniques, namely the Double-Row Suture Anchor Fixation (DR) and Suture-Bridge Technique (SB), were used to repair humeral GT fractures in 12 fresh-frozen human cadaveric shoulders. A Tekscan pressure sensor placed between the repaired tuberosity and humerus recorded continuous data points directly after repair and for 60 min at set time intervals. The constructs were then cyclically loaded until 100 N, and the shoulders tested at 0°, 30°, and 60° of abduction. Under an applied force, the contact pressure and contact area of the interface were determined.

**Results:**

Although both fixation configurations showed decreased contact pressure and area over time, the SB group had higher contact pressure right after fixation and at all time points thereafter. In contrast, the DR group demonstrated significantly more contact pressure and area at each abduction position with the applied load. Nevertheless, contact pressure and area decreased in response to increasing abduction position for both fixation constructs.

**Conclusion:**

Findings suggest that despite the SB construct having superior interface contact immediately after fixation, the DR construct offered better contact performance at all abduction angles with applied force.

**Level of evidence:**

Basic Science, Biomechanics.

## Background

Good results have been reported with suture anchor fixations of humeral greater tuberosity (GT) fractures [1–8]. Arthroscopic or open fixation using suture anchors with double-row constructs improves the initial repair strength and may provide a stable interface suitable to biologically heal and reestablish the normal anatomy [9]. However, biomechanical data regarding the fixation method of GT fractures are scarce. One previous study compared the biomechanical strength of three fixation constructs that use either suture anchors or screws. The two double-row suture anchor fixations showed comparable loading force for a 5 mm fracture displacement and failure load, and both methods achieved superior results in load-gap formation and failure load compared with the two-screw fixation [9]. To our knowledge, no study has been conducted to investigate the contact characteristics, including contact pressure and area, at the fracture interface with different suture anchor fixation constructs for GT fracture. We believe there may be a suture anchor fixation construct for GT fracture that optimizes the contact characteristics [10–13].

In addition, an abduction brace and related exercises are routinely used after humeral GT fracture repair [14, 15]. Enhancing the biomechanical properties of the GT repair enables patients to regain mobility soon after surgery without reduction loss or fixation failure. Lin et al. suggested that shoulder abduction can affect the fixation strength of humeral GT fracture. When the double-row suture anchor construct and suture bridge technique are used in combination, superior biomechanical properties at various abduction angles can be achieved [15]. However, no biomechanical studies have explored the effects of shoulder-abduction angle on the contact pressure and contact area at the fracture interface of the repaired GT fracture. Moreover, the effects on contact pressure and area are clinically pertinent if the biomechanical properties after surgery are to be optimized.

The predicted healing time was strongly influenced by the fixation stability and in particular by the increased interfragmentary shear movements [16, 17]. Previous studies indicate the important role of the initial mechanical stability specifically in the vascularization of an osteosynthesis [18]. Improving fixation stability is crucial to optimal fracture healing. Accordingly, we conducted this cadaveric study to investigate: (1) the contact pressure and contact area at the fracture interface of two double-row fixation techniques, both initially after fixation and over time; and, (2) the contact pressure and contact area at the fracture interface under cyclic loading of the rotator cuff tendon at the abduction angles of 0^o^, 30^o^ and 60^o^. This study analyzed the fracture contact properties of double-row suture anchor fixations for GT fracture as well as the effect of shoulder abduction on fracture interface contact. We hypothesized that contact strength will reduce over time and different suture anchor constructs will have different contact properties in response to an applied force and shoulder abduction position.

## Methods

### Specimen preparation

The institutional review board assessed and sanctioned this study. Twelve fresh-frozen human cadaveric shoulder specimens (mean age of 60.7+ 2.1 years) kept at -20 °C, without gross evidence of rotator cuff injury or GT bone cyst, were used for this study. Samples were slowly defrosted to 25 °C for ~ 24 h before dissection and testing. Bone mineral density was measured on the greater tuberosity using dual energy X-ray absorptiometry (DXA) to assess bone quality at the site of the fracture interface prior to testing. Aside from the supraspinatus tendon, all soft tissues were resected from the scapula and humerus, with only the whole humerus and supraspinatus tendon retained. A length of over 3 cm of the supraspinatus tendon was preserved at the bony insertion. After cutting the distal humeral condyle, a humeral shaft length of 20 cm remained. Specimens were kept moist during all phases of dissection, preparation, and testing using normal saline solution. Standardized osteotomies oriented 50^o^ with respect to the humeral shaft were performed at the base of the GT using a thin-blade reciprocating saw. The osteotomy was initiated 2 mm medial to the footprint of the supraspinatus tendon and directly posterior to the bicipital groove. Specimens were randomly apportioned to either the suture bridge repair group (SB group) (*n* = 6) or double-row suture anchor repair group (DR group) (n = 6). A single orthopedic surgeon completed all fixation constructs for all samples.

### Fixation configurations

Two types of fixation were performed in this study as follows: (1) suture bridge fixation, and (2) double-row suture anchor fixation [9].

#### Suture-bridge fixation (SB group) (Fig. 1a)

Two single loaded medial-row suture anchors (Corkscrew, 5.0 mm, Arthrex, Naples, FL, USA) were placed at an orientation of 45° (dead man’s angle) on the articular margin of the humeral head. The second medial anchor was implanted 1.5 cm posterior to the first one, after which the sutures (No. 2 FiberWire, Arthrex) were threaded through the intact cuff connected to the GT fragments as a mattress suture. The medial mattress sutures were tied first. Two pilot holes for the knotless suture anchors, PushLock (3.5 mm, Arthrex, Naples, FL, USA), were precisely aligned with the medial anchors and approximately 5 mm distal to the lateral edge of the GT fragment. A suture limb from each medial suture anchor was then passed through the PushLock eyelet on the distal end of the driver. With continuous applied force, two PushLock anchors were implanted into the pilot holes using the suture-bridge technique.

**Fig. 1:**
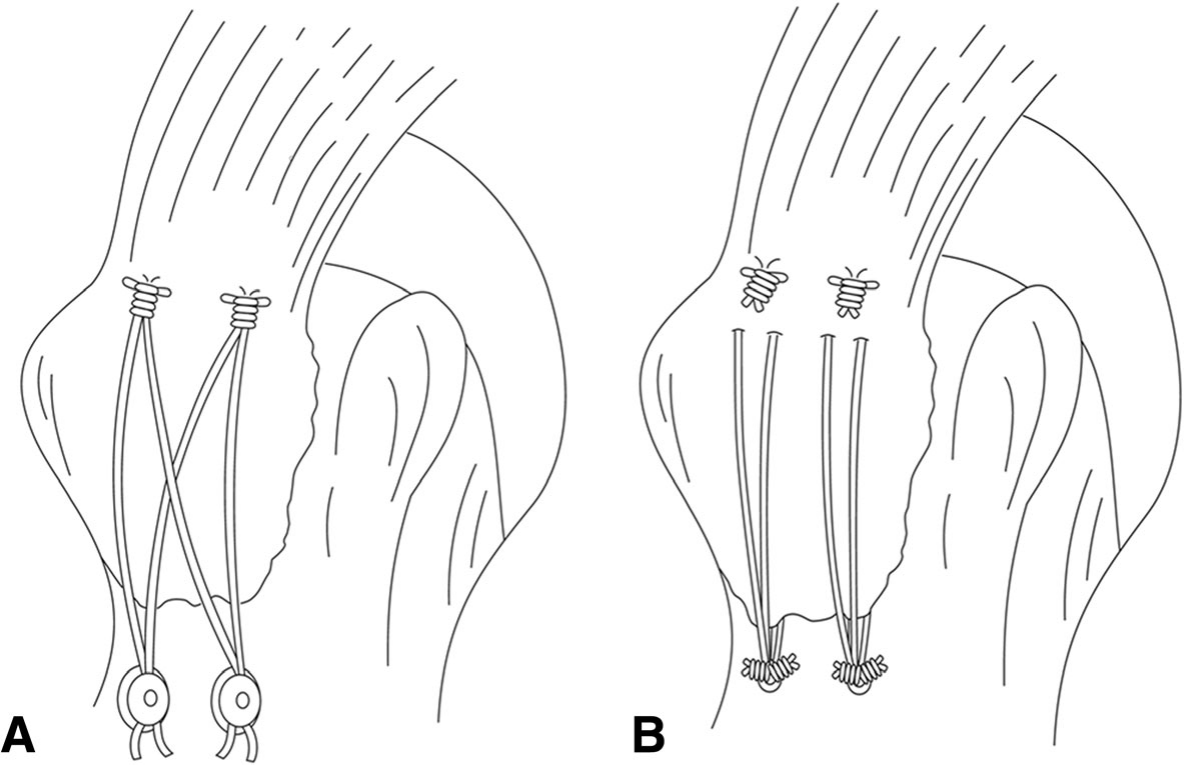
Two double-row fixation configurations. **a** Double-row suture anchor fixation. **b** Suture bridge technique

#### Double-row suture anchor fixation (DR group) (Fig. 1b)

The medial row was the same as the above. Two double-loaded lateral-row suture anchors (Corkscrew, 5.0 mm, Arthrex, Naples, FL, USA) were then inserted into the cortex of the humerus approximately 5 mm distal to the lateral edge of the GT fragment. One limb from both sutures (No. 2 FiberWire) of each lateral-row anchor was threaded through the intact cuff between the medial row of the sutures and the medial aspect of the fracture fragments. The medial-row sutures were then knotted as a mattress suture, followed by the four strands of the two lateral-row anchors knotted as simple sutures.

### Pressure sensory preparation

**Fig. 2:**
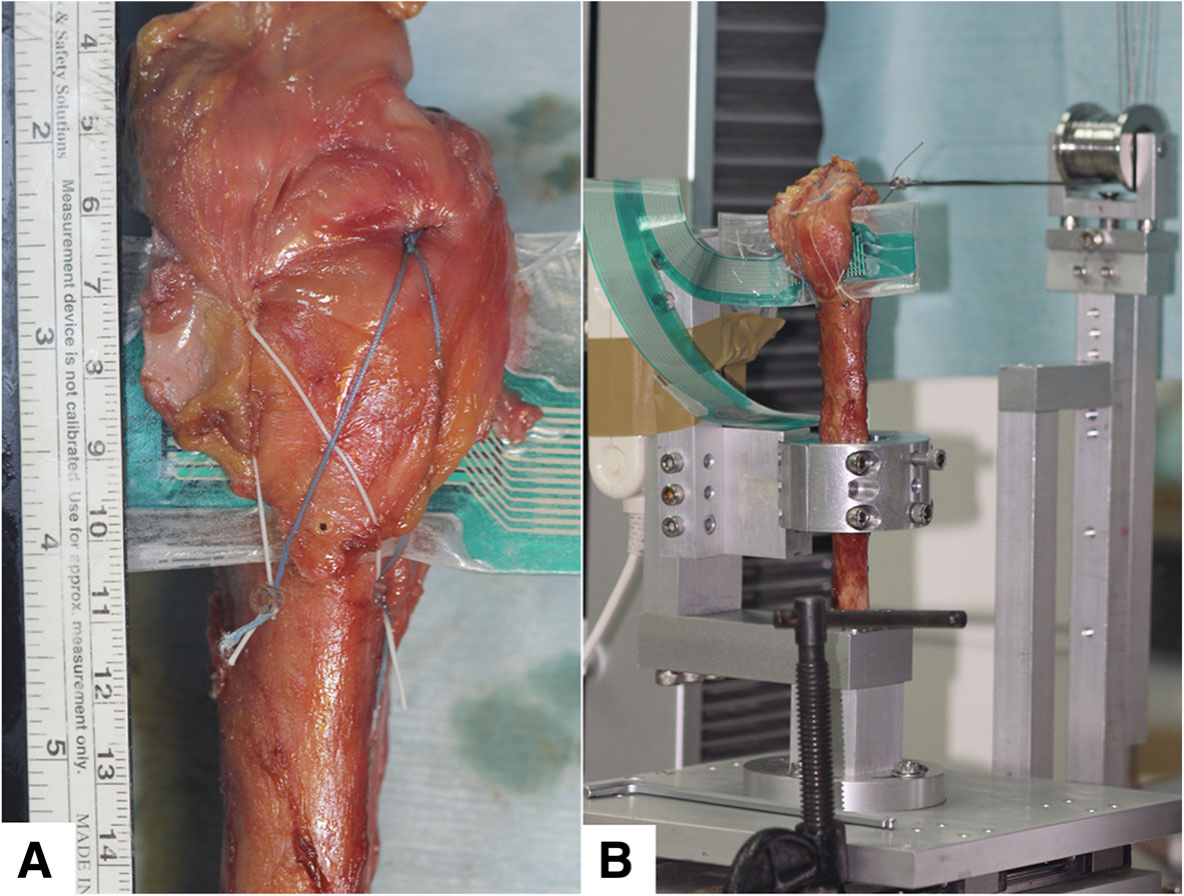
**a** Pressure sensor preparation. **b** Mechanical testing setup

Measurements of the contact pressure and area between the GT-fragment interface and its underling humerus fracture site for each specimen were performed using a Tekscan I-Scan System (Tekscan,Inc., South Boston, Massachusetts). TekScan sensors are highly accurate and can collect data points continuously in real time, thereby allowing the fixation techniques to be studied over time [19]. To increase accuracy and maximize the potential interface contact area within the suture-anchor constructs, the TekScan pressure pads were individually tailored to match the shape of the GT fragment before testing. Because the working area was larger than the average fracture area, the Tekscan sensors could be embedded in our fixations while maintaining data-recording sensitivity. Clear waterproof tape smoothed with a seam-roller was employed to ensure the sensor was hermetically sealed (Fig. 2a). It should be noted that the pilot study indicated that neither the sensitivity nor repeatability of the sensor measurements were impacted by the sealing process. Installation of the Tekscan sensors was performed as described herein. Although the medial anchors were positioned first and the sutures were either tied (for SB group) or threaded (for DR group), the medial aspect of the GT and the sensor were both incorporated. Then, a Tekscan sensor was fitted at the interface of the osteotomy. After tensioning the sutures laterally over the GT edge, the sutures were fixed into bone (SB group) or tired securely (DR group). All pressure sensors were calibrated before testing using a material testing machine load cell (MTS, AG-X, Shimadzu Corp., Tokyo, Japan) with the Tekscan sensitivity set to “high”. Subsequent to all specimen measurements, each sensor was detached and its functionality and sensitivity re-verified via the MTS load cell.

### Biomechanical testing

The humeral shaft was secured to the customized fixture mounted upright on the extended plate coupled to the working surface of the universal material testing machine (MTS, AG-X, Shimadzu Corp., Tokyo, Japan). The supraspinatus tendons were gripped using a Krackow method with a stainless steel wire attached onto the myotendinous junction. The cable wire was then passed through a pulley fastened to the working surface of the MTS. We have previous established this model of humeral GT fracture and described it in the literature [9] (Fig. 2b).

The contact pressure and area measurements were recorded immediately after the GT-fracture fixation without applied force. Additional data points were recorded every minute for the first 10 min, then every 5 min for the following 20 min, and lastly every 10 min for the final 30 min, for a total of 18 data points spread over the total data collection period of 60 min.

Subsequently, the applied traction force was directed to the abduction angles of 0°, 30° and 60°. The abduction angle was defined as the traction cable relative to the horizontal plane [15]. The pilot study revealed that the abduction-position order during testing did not affect the results. A 20 N preload was set, after which all fixations were cyclically loaded with 40 N for 10 cycles; then, the load was increased to 100 N over 10 cycles in 20 N increments. These abduction positions with applied force were chosen to simulate a postoperative rehabilitation regimen, with the pulling speed set to 60 mm/min.

### Statistical analysis

Statistical analysis was performed with SPSS software (version 17.0; SPSS Inc., Chicago, IL, USA). The Mann-Whitney U test was conducted to compare the bone mineral density, fracture contact area, and contact pressure with pre-load and cyclic loading at 100 N between the 2 fixation construct groups. The Kruskal-Wallis test with the Student-Newman-Keuls post-hoc test was used to evaluate differences between the 3 abduction positions (0^o^, 30^o^ and 60^o^) for both fixation constructs. Statistical significance was set at *P* < 0.05. A post hoc power analysis was also performed to ensure there were enough specimens in each test group to detect all statistical differences and avoid type II error. The results established that a sample size of at least 6 specimens per group would provide a study power of 0.8 and an α value of 0.05.

## Results

The bone mineral density did not statistically vary between the two tested groups, averaging 0.69 + 0.06 g/cm^2^ in the SB group and 0.68 + 0.05 g/cm^2^ in the SB group (*p = 0.988*).

### Initial fixation properties and changes over time

**Fig. 3:**
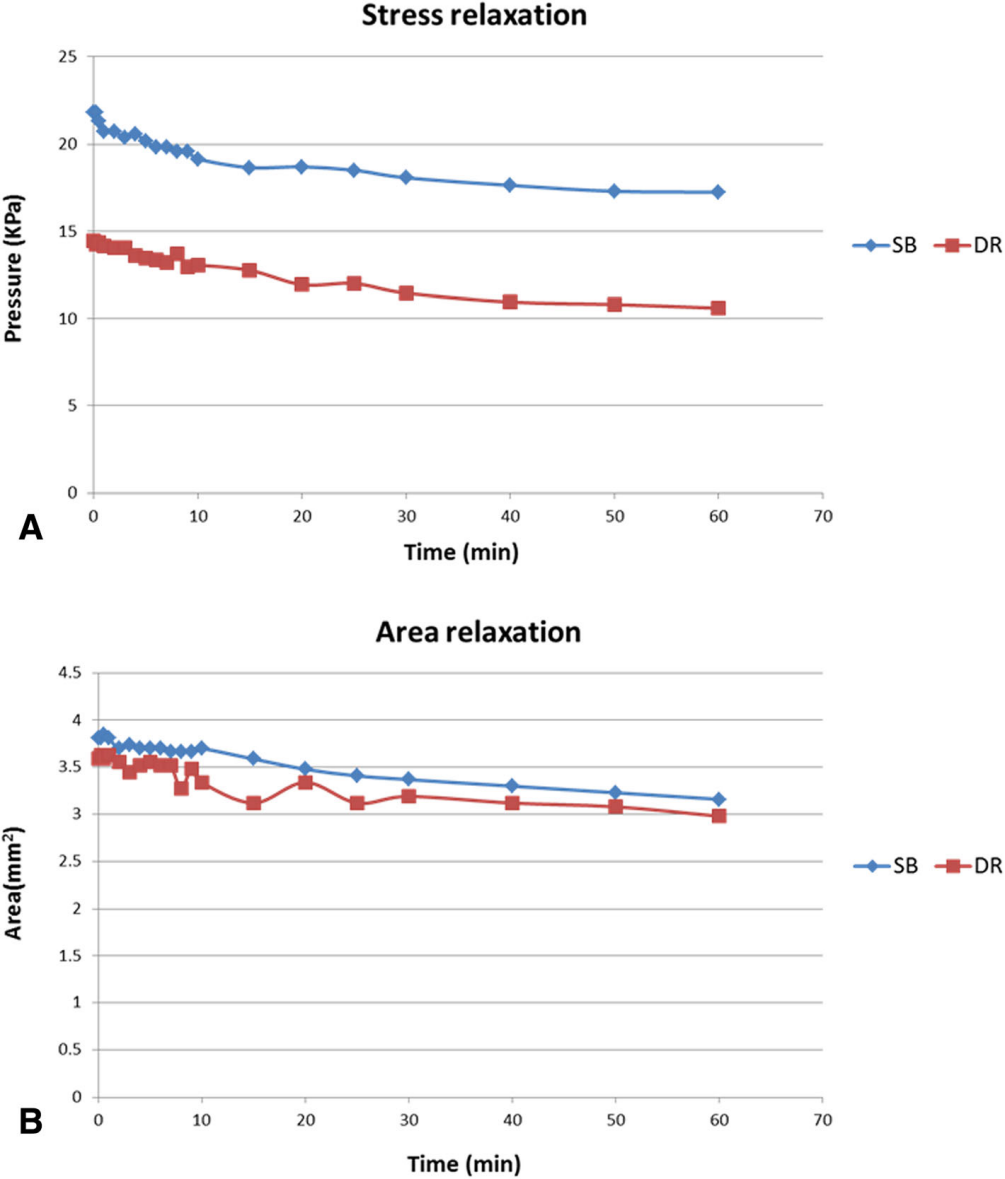
**a** Stress relaxation over time. **b** Area relaxation over time

Results of the mean contact pressure and area immediately after fixation and the changes over time are shown in Fig. 3. As can be seen, both groups showed reductions in contact parameters over time (stress and area relaxation). The SB group had a higher mean contact pressure than the DR group immediately after fixation (21.8 vs.14.5 Kpa, *p =* 0.038), as well as at all data points over the 60-min data collection period. The SB and DR groups had 21 and 27% drops, respectively, in contact pressure over the 60-min period (17.3 vs.10.6 Kpa, *p =* 0.027). With respect to the mean contact area, no statistically significant differences were found between the SB and DR groups immediately after fixation or at any data point throughout the 60 min. Nevertheless, both groups registered a 17% drop in the mean contact area over the testing period.

### Mean contact pressure and contact area in response to applied load under different abduction angles

**Fig. 4:**
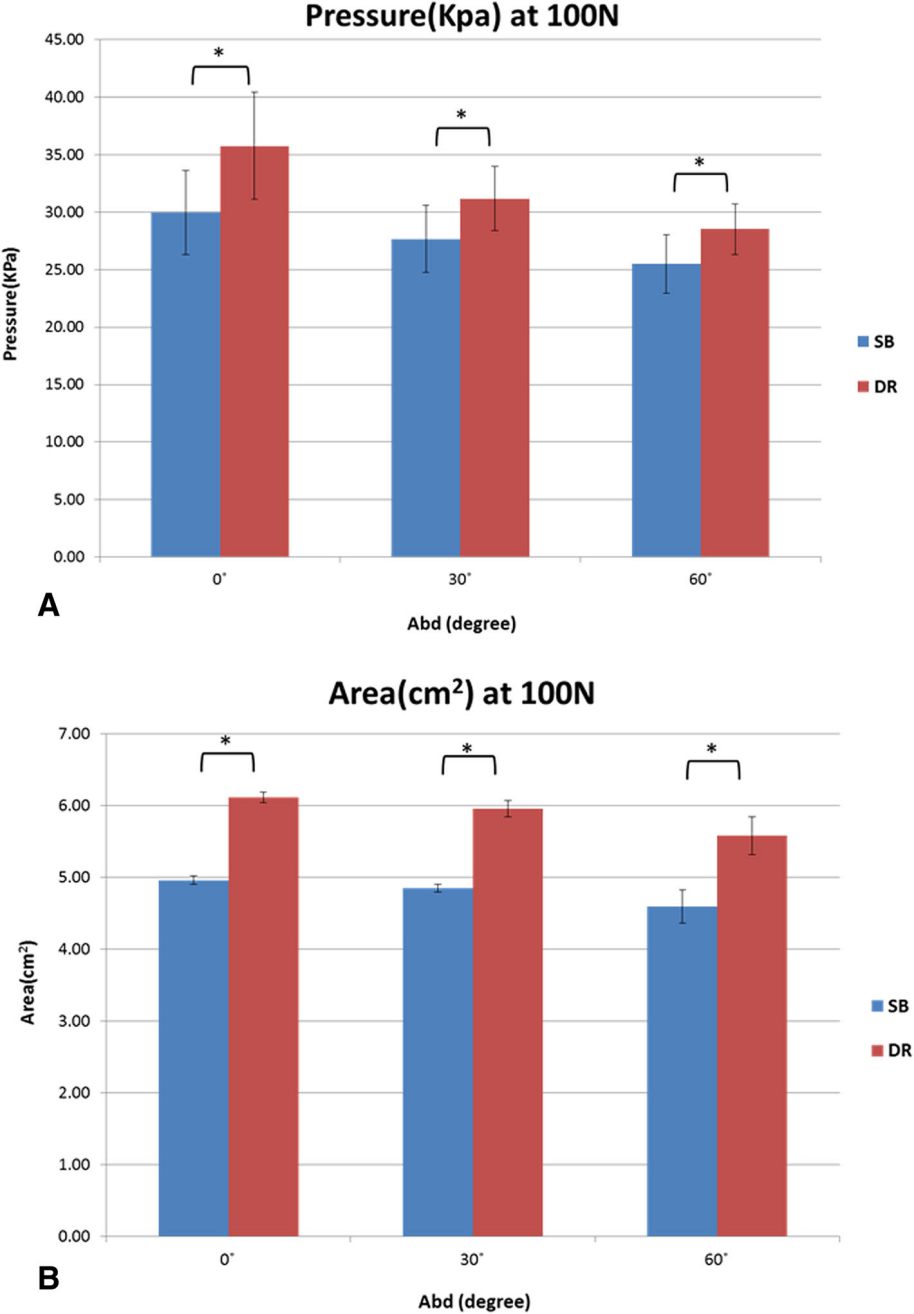
**a** Contact pressure at 100 N between SB and DR groups for abduction angles of 0^o^, 30^o^ and 60^o^. **b** Contact area at 100 N between SB and DR groups for abduction angles of 0^o^, 30^o^ and 60^o^

The mean interface pressure exerted on the humerus by the GT was greater for the DR group compared with the SB group at each abduction angle in response to the applied 100 N load (Fig. 4a). The mean interface pressures exerted over the fracture site by the GT were 29.9 + 3.7 Kpa at 0^o^, 27.7 + 2.9 Kpa at 30^o^, and 25.5 + 2.5 Kpa at 60^o^ for the SB group, and 35.8+ 4.6Kpa at 0^o^, 31.2+ 2.8Kpa at 30^o^, and 28.5+ 2.2Kpa at 60^o^ for the DR group.

At each abduction angle, the DR construct also provided a larger fracture contact area than the SB construct under the 100 N load (Fig. 4b). The mean interface contact areas were 4.96+ 0.06 cm^2^ at 0^o^, 4.85 + 0.05 cm^2^ at 30^o^, and 4.59 + 0.23 cm^2^ at 60^o^ for the SB group, and 6.11 + 0.08 cm^2^ at 0^o^, 5.95 + 0.11 cm^2^ at 30^o^, and 5.58 + 0.26 cm^2^ at 60^o^ for the DR group. A comparison of the two constructs for both contact area and pressure showed that the DR group provided significantly more contact area at each tested position.

**Fig. 5:**
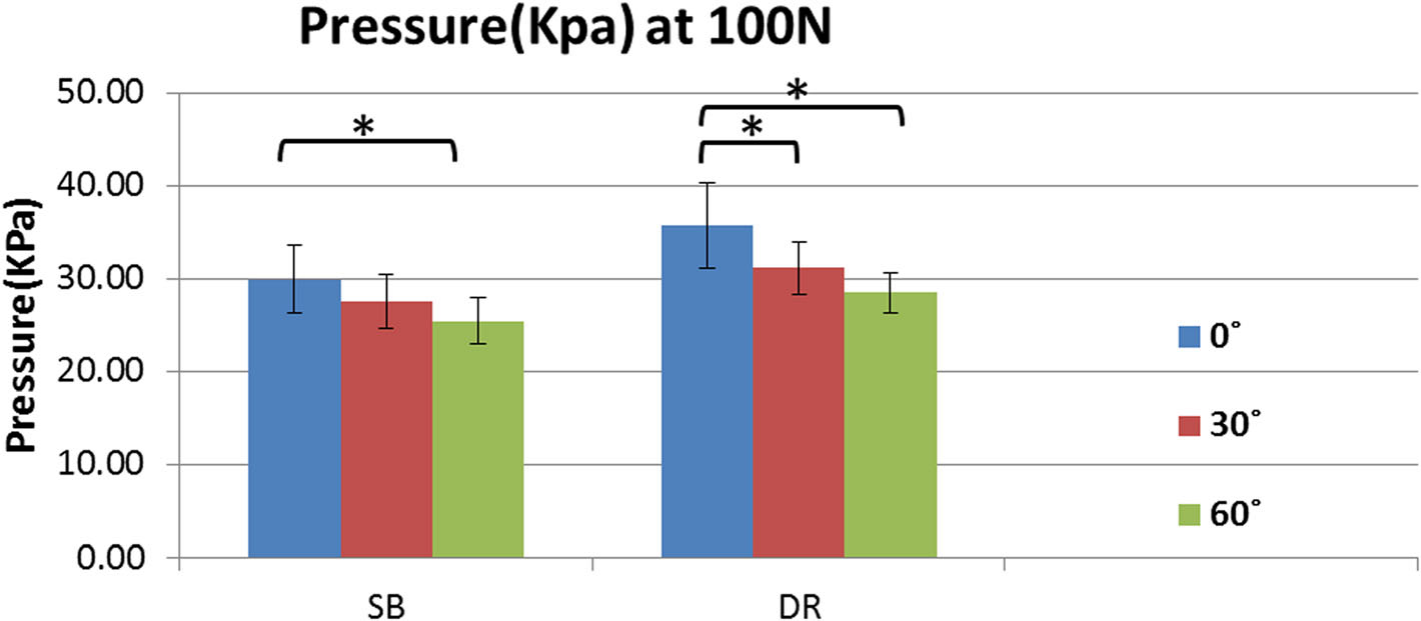
Comparison of contact pressure at 100 N among the different abduction angles

The highest mean interface pressure exerted over the fracture site by the GT was achieved at 0° of abduction for both the SB and DR groups (29.9Kpa and 35.8Kpa, respectively). Moreover, it was found that the SB group had a significant decrease in contact pressure between 0° to 60° of abduction; meanwhile, the DR group had significant decreases in overall fracture contact pressure from 0° to 30° and 0° to 60° of abduction. All other contact pressure comparisons among the different abduction positions were not significant (Table 1 and Fig. 5).

**Table 1:** Contact Pressure Comparisons for Suture-Bridge and Double-Row Suture Anchor fixations at 0°, 30°, and 60° of Abduction

			contact pressure comparisons, Kpa						
Fixation	0°	30°	*p*	30°	60°	*p*	0°	60°	*p*
Suture-Bridge	29.9	27.7	0.209	27.7	25.5	0.24	29.9	25.5	**0.023**
Double-Row	35.8	31.2	**0.032**	31.2	28.5	0.194	35.8	28.5	**0.004**

Boldface: *p* < 0.05

**Fig. 6:**
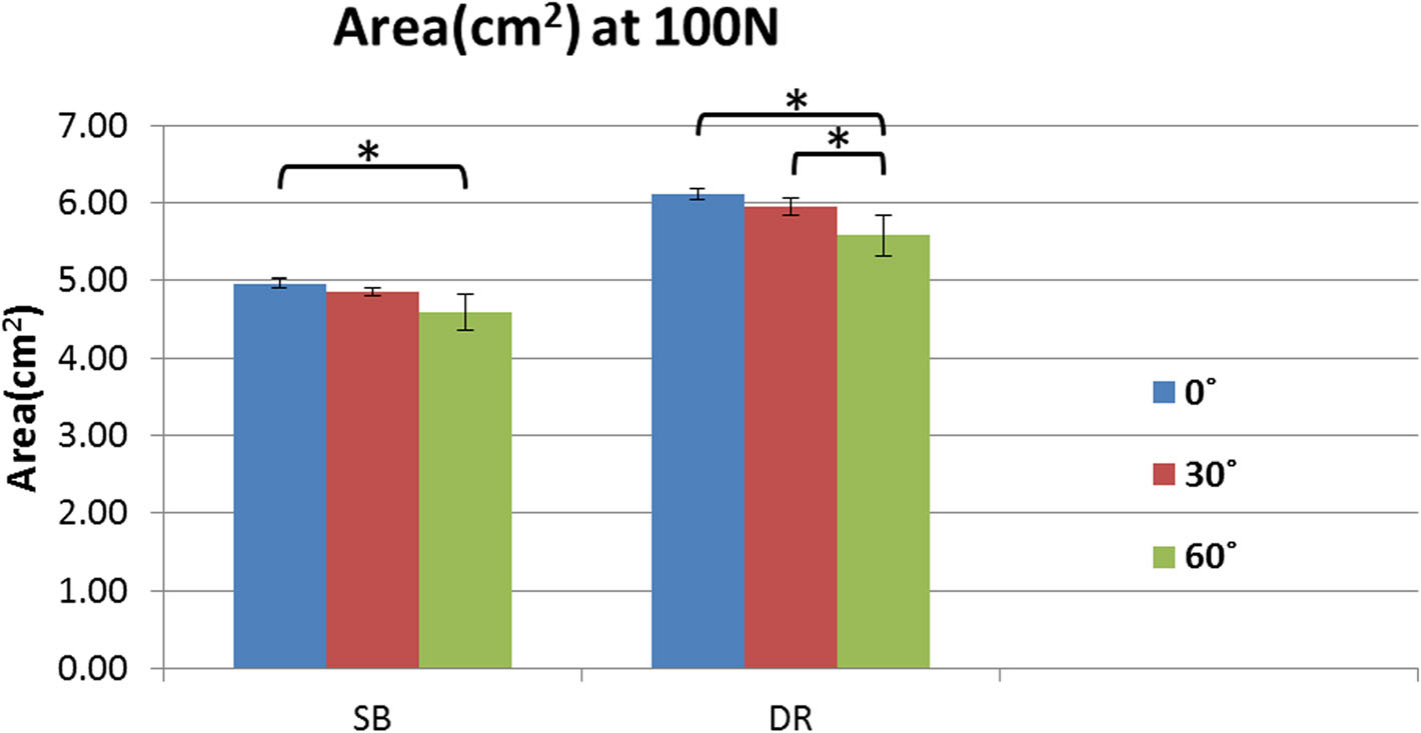
Comparison of contact area at 100 N among the different abduction angles

Similarly, the mean pressurized contact area at the tuberosity fracture site was also highest at 0° of abduction for both the SB and DR groups (4.96 cm^2^ and 6.11 cm^2^, respectively). The SB group experienced a significant reduction in contact area from 0° to 60° of abduction, while for the DR group, abduction from 0° to 60° and 30° to 60° significantly decreased overall fracture contact area. All other contact area comparisons among different abduction positions were not significant (Table 2 and Fig. 6).

**Table 2:** Contact Area Comparisons for Suture-Bridge and Double-Row Suture Anchor fixations at 0°, 30°, and 60° of Abduction

			contact area comparisons, cm^2^						
Fixation	0°	30°	*p*	30°	60°	*p*	0°	60°	*p*
Suture-Bridge	4.96	4.85	0.524	4.85	4.59	0.144	4.96	4.59	**0.044**
Double-Row	6.11	5.95	0.363	5.95	5.58	**0.039**	6.11	5.58	**0.006**

Boldface: *p* < 0.05

## Discussion

In this cadaveric model, both double-row suture anchor constructs showed decreased contact pressure and area over time. Further, the SB construct had greater contact pressure at all time points compared with the DR fixation. The current study also sought to characterize the interface contact properties in various shoulder abduction positions under applied forces. The results suggest that the DR group provided significantly more contact pressure and area at each tested abduction position. In addition, abduction from a low to high angle decreased fracture contact pressure and area for both fixation methods. To our best knowledge, this study is the first to investigate the contact features for the fixation of GT fractures.

Although several studies have investigated the biomechanical strength of GT fixation constructs, the results reflected only the strength immediately following fixation, not over time [9, 20–23]. In response, our report indicated that both the SB and DR constructs had the phenomenon of “stress and area relaxation” over time. These findings may be explained in that given the intimate association of the rotator cuff with the GT, the double-row suture anchor constructs were incorporated into the rotator cuff tendons. More specifically, the viscoelastic properties of the tendons and the suture materials would lead to reductions of the interface contact. A similar phenomenon is also observed in rotator cuff repair [13]. Be that as it may, more studies are needed to further clarify the clinical significance of stress and area relaxation.

**Fig. 7:**
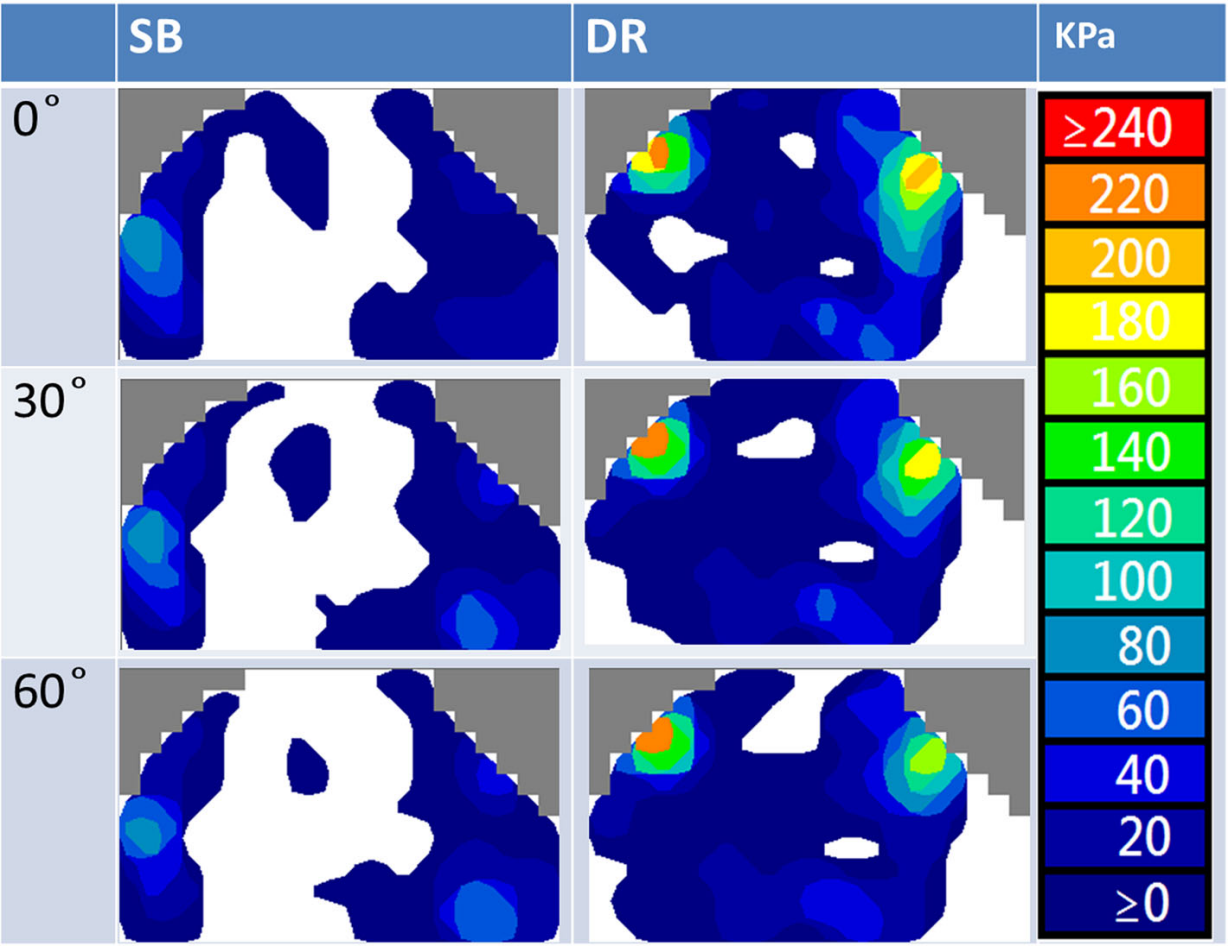
Example of contact pressure and area with applied force

Different contact properties in different repair constructs have been reported in several rotator cuff studies [11, 12, 24–27]. Some have focused on the contact properties between the SB and DR constructs in rotator cuff repair. Although two studies by Park et al. [11, 26] showed that the SB construct had higher contact pressure and area than the DR construct, Ostrander et al. [24] reported that no statistical difference in contact pressure or contact area could be found between the SB and DR techniques. Regarding the GT fixation in this study, the SB group had higher contact pressure than the DR group immediately after fixation. This might be attributable to the nature of the fixation construct itself, which is similar to that used in rotator cuff studies [11, 26]. When the 100 N load was applied to the construct, results showed that the DR group had significantly more contact pressure and contact area than the SB group. We speculate that the contact properties may be determined by both the fixation construct as well as how the construct converts the applied load into compression force on the GT fragment. The current results suggest that the DR construct was more effective in transforming the applied force into compression force (Fig. 7), the mechanism of which is akin to the tension bands configuration [28]. However, further studies are needed to identify the exact mechanism of force conversion in these constructs.

A previous biomechanical study indicated that double-row suture anchor constructs have different biomechanical performances at different abduction positions after GT fracture fixation [15]. Adding to this, the current study revealed that the interface contact pressure and area were greater for the DR technique compared with SB technique at each abduction angle in response to an applied 100 N load. As aforementioned, the DR construct might be more effective in transforming applied force into compression force. Nevertheless, both the SB and DR constructs had decreased contact pressure and area when tested at increased abduction angles. A possible explanation is that the direction of applied force becomes more parallel to the fracture interface with increasing abduction angle, thereby converting the applied force into shear force and decreasing the interface contact. Accordingly, changes in these parameters should be taken into consideration for rehabilitation regimens and fracture healing [16, 17].

Although isolated fractures of the humeral GT are less common than three- or four-part fractures of the proximal humerus, they can still result in significant disability. Moreover, the intimate association of the rotator cuff with the tuberosities has a substantial impact on these injuries. When fractured, the greater tuberosity often displaces posteriorly and superiorly due to the deforming forces of the rotator cuff muscles. If its displacement is more than 5 mm, it can affect overhead elevation of the arm and cause subacromial impingement, and may block external rotation with the arm at the side [29, 30]. Arthroscopic and open fixation techniques can be used to surgically treat GT fracture with good patient outcomes [30, 31]. Moreover, our results will aid evidence-based medicine in decision-making for treating this unique fracture, particularly when surgical intervention with suture anchor-based fixation is considered.

The results in our report are limited by the use of an in vitro cadaveric model. As such, postoperative fracture healing was not taken into account, and so the contact properties when healing starts are not reflected. Furthermore, our model lacked certain pertinent forces, including those of the infraspinatus and teres minor, which may affect the clinical significance of GT fractures. As such, common to all such models, our study utilized a fracture model created in a lab environment that does not account for clinical variables such as fracture comminution, size of GT fragment, degree of trauma to the rotator cuff and injury to other soft tissues. However, being a comparative study, both groups investigated were subject to the same limitations, and so the general findings should be unaffected. Accordingly, we assume that our results can provide pertinent information regarding the contact characteristics of these two double-row suture anchor fixation techniques.

## Conclusion

In conclusion, the SB construct had better initial contact pressure than the DR construct immediately after fixation and over time. However, with an applied force, the DR fixation provided significantly more contact pressure and area at each abduction position tested. With increasing abduction angle, decreased fracture contact pressure and area for both fixation methods were noted. These results suggest that although the SB construct provides better interface contact initially after fixation, the DR construct would offer superior contact performance during early rehabilitation programs with abduction exercise. Nevertheless, caution is still advised when conducting abduction-exercise regimens after suture anchor-based fixation.

## Abbreviations

DR: Double-row
GT: Greater tuberosity
MTS: Material testing machine
SB: Suture-Bridge

## References

[1] Bhatia DN, van Rooyen KS, du Toit DF, de Beer JF (2006). Surgical treatment of comminuted, displaced fractures of the greater tuberosity of the proximal humerus: a new technique of double-row suture-anchor fixation and long-term results. Injury.

[2] Ji JH, Kim WY, Ra KH (2007). Arthroscopic double-row suture anchor fixation of minimally displaced greater tuberosity fractures. Arthroscopy.

[3] Ji JH, Moon CY, Kim YY, Shafi M (2009). Arthroscopic fixation for a malunited greater tuberosity fracture using the suture-bridge technique: technical report and literature review. Knee Surg Sports Traumatol Arthrosc.

[4] Cadet ER, Ahmad CS (2007). Arthroscopic reduction and suture anchor fixation for a displaced greater tuberosity fracture: a case report. J Shoulder Elb Surg.

[5] Kim KC, Rhee KJ, Shin HD, Kim YM (2008). Arthroscopic fixation for displaced greater tuberosity fracture using the suture-bridge technique. Arthroscopy.

[6] Ji JH, Shafi M, Song IS, Kim YY, McFarland EG, Moon CY (2010). Arthroscopic fixation technique for comminuted, displaced greater tuberosity fracture. Arthroscopy.

[7] Song HS, Williams GR, Jr.: Arthroscopic reduction and fixation with suture-bridge technique for displaced or comminuted greater tuberosity fractures. Arthroscopy 2008, 24(8):956–960. 10.1016/j.arthro.2008.01.009 18657746

[8] Liao W, Zhang H, Li Z, Li J (2016). Is arthroscopic technique superior to open reduction internal fixation in the treatment of isolated displaced greater tuberosity fractures?. Clin Orthop Relat Res.

[9] Lin CL, Hong CK, Jou IM, Lin CJ, Su FC, Su WR (2012). Suture anchor versus screw fixation for greater tuberosity fractures of the humerus--a biomechanical study. J Orthop Res.

[10] Grimberg J, Diop A, Kalra K, Charousset C, Duranthon LD, Maurel N (2010). In vitro biomechanical comparison of three different types of single- and double-row arthroscopic rotator cuff repairs: analysis of continuous bone-tendon contact pressure and surface during different simulated joint positions. J Shoulder Elb Surg.

[11] Park MC, Pirolo JM, Park CJ, Tibone JE, McGarry MH, Lee TQ (2009). The effect of abduction and rotation on footprint contact for single-row, double-row, and modified double-row rotator cuff repair techniques. Am J Sports Med.

[12] Kim SJ, Kim SH, Moon HS, Chun YM (2016). Footprint contact area and Interface pressure comparison between the knotless and knot-tying Transosseous-equivalent technique for rotator cuff repair. Arthroscopy.

[13] Mazzocca AD, Bollier MJ, Ciminiello AM, Obopilwe E, DeAngelis JP, Burkhart SS, Warren RF, Arciero RA (2010). Biomechanical evaluation of arthroscopic rotator cuff repairs over time. Arthroscopy.

[14] Gruson KI, Ruchelsman DE, Tejwani NC (2008). Isolated tuberosity fractures of the proximal humeral: current concepts. Injury.

[15] Lin CL, Su FC, Chang CH, Hong CK, Jou IM, Lin CJ, Su WR (2015). Effect of shoulder abduction on the fixation of humeral greater tuberosity fractures: a biomechanical study for three types of fixation constructs. J Shoulder Elb Surg.

[16] Wehner T, Claes L, Niemeyer F, Nolte D, Simon U (2010). Influence of the fixation stability on the healing time--a numerical study of a patient-specific fracture healing process. Clin Biomech (Bristol, Avon).

[17] Schell H, Epari DR, Kassi JP, Bragulla H, Bail HJ, Duda GN (2005). The course of bone healing is influenced by the initial shear fixation stability. J Orthop Res.

[18] Lienau J, Schell H, Duda GN, Seebeck P, Muchow S, Bail HJ (2005). Initial vascularization and tissue differentiation are influenced by fixation stability. J Orthop Res.

[19] Drewniak EI, Crisco JJ, Spenciner DB, Fleming BC (2007). Accuracy of circular contact area measurements with thin-film pressure sensors. J Biomech.

[20] Gaudelli C, Menard J, Mutch J, Laflamme GY, Petit Y, Rouleau DM (2014). Locking plate fixation provides superior fixation of humerus split type greater tuberosity fractures than tension bands and double row suture bridges. Clin Biomech (Bristol, Avon).

[21] Brais G, Menard J, Mutch J, Laflamme GY, Petit Y, Rouleau DM (2015). Transosseous braided-tape and double-row fixations are better than tension band for avulsion-type greater tuberosity fractures. Injury.

[22] Braunstein V, Wiedemann E, Plitz W, Muensterer OJ, Mutschler W, Hinterwimmer S (2007). Operative treatment of greater tuberosity fractures of the humerus--a biomechanical analysis. Clin Biomech (Bristol, Avon).

[23] Seppel G, Saier T, Martetschlager F, Plath JE, Guevara-Alvarez A, Henschel J, Winkler M, Augat P, Imhoff AB, Buchmann S (2017). Single versus double row suture anchor fixation for greater tuberosity fractures - a biomechanical study. BMC Musculoskelet Disord.

[24] Ostrander RV, McKinney BI (2012). Evaluation of footprint contact area and pressure using a triple-row modification of the suture-bridge technique for rotator cuff repair. J Shoulder Elb Surg.

[25] Tuoheti Y, Itoi E, Yamamoto N, Seki N, Abe H, Minagawa H, Okada K, Shimada Y (2005). Contact area, contact pressure, and pressure patterns of the tendon-bone interface after rotator cuff repair. Am J Sports Med.

[26] Park MC, ElAttrache NS, Tibone JE, Ahmad CS, Jun BJ, Lee TQ (2007). Part I: footprint contact characteristics for a transosseous-equivalent rotator cuff repair technique compared with a double-row repair technique. J Shoulder Elb Surg.

[27] Park MC, Cadet ER, Levine WN, Bigliani LU, Ahmad CS (2005). Tendon-to-bone pressure distributions at a repaired rotator cuff footprint using transosseous suture and suture anchor fixation techniques. Am J Sports Med.

[28] Brink PR, Windolf M, de Boer P, Brianza S, Braunstein V, Schwieger K (2013). Tension band wiring of the olecranon: is it really a dynamic principle of osteosynthesis?. Injury.

[29] Bono CM, Renard R, Levine RG, Levy AS (2001). Effect of displacement of fractures of the greater tuberosity on the mechanics of the shoulder. J Bone Joint Surg Br.

[30] DeBottis D, Anavian J, Green A (2014). Surgical management of isolated greater tuberosity fractures of the proximal humerus. Orthop Clin North Am.

[31] Rouleau DM, Mutch J, Laflamme GY (2016). Surgical treatment of displaced greater tuberosity fractures of the Humerus. J Am Acad Orthop Surg.

